# Using trials of caloric restriction and bariatric surgery to explore the effects of body mass index on the circulating proteome

**DOI:** 10.1038/s41598-023-47030-x

**Published:** 2023-11-29

**Authors:** Lucy J. Goudswaard, Madeleine L. Smith, David A. Hughes, Roy Taylor, Michael Lean, Naveed Sattar, Paul Welsh, Alex McConnachie, Jane M. Blazeby, Chris A. Rogers, Karsten Suhre, Shaza B. Zaghlool, Ingeborg Hers, Nicholas J. Timpson, Laura J. Corbin

**Affiliations:** 1 Population Health Sciences, University of Bristol, Oakfield House, Oakfield Grove, Bristol, BS8 2BN UK; 2 MRC Integrative Epidemiology Unit, Bristol, UK; 3 Physiology, Pharmacology & Neuroscience, University of Bristol, Biomedical Sciences Building, University Walk, Bristol, BS8 1TD UK; 4 Newcastle Magnetic Resonance Centre, Translational and Clinical Research Institute, Newcastle University, Newcastle Upon Tyne, NE4 5PL UK; 5 Human Nutrition, School of Medicine, Dentistry and Nursing, College of Medical, Veterinary & Life Sciences, University of Glasgow, Glasgow, G31 2ER UK; 6 School of Cardiovascular and Medical Science, University of Glasgow, Glasgow, G12 8TA UK; 7 Robertson Centre for Biostatistics, School of Health and Wellbeing, University of Glasgow, Glasgow, G12 8QQ UK; 8 Bristol Medical School, Bristol Trials Centre, University of Bristol, Bristol, BS8 1NU UK; 9 Department of Biophysics and Physiology, Weill Cornell Medicine - Qatar, Doha, Qatar

**Keywords:** Obesity, Risk factors, Epidemiology

## Abstract

Thousands of proteins circulate in the bloodstream; identifying those which associate with weight and intervention-induced weight loss may help explain mechanisms of diseases associated with adiposity. We aimed to identify consistent protein signatures of weight loss across independent studies capturing changes in body mass index (BMI). We analysed proteomic data from studies implementing caloric restriction (Diabetes Remission Clinical trial) and bariatric surgery (By-Band-Sleeve), using SomaLogic and Olink Explore1536 technologies, respectively. Linear mixed models were used to estimate the effect of the interventions on circulating proteins. Twenty-three proteins were altered in a consistent direction after both bariatric surgery and caloric restriction, suggesting that these proteins are modulated by weight change, independent of intervention type. We also integrated Mendelian randomisation (MR) estimates of the effect of BMI on proteins measured by SomaLogic from a UK blood donor cohort as a third line of causal evidence. These MR estimates provided further corroborative evidence for a role of BMI in regulating the levels of six proteins including alcohol dehydrogenase-4, nogo receptor and interleukin-1 receptor antagonist protein. These results indicate the importance of triangulation in interrogating causal relationships; further study into the role of proteins modulated by weight in disease is now warranted.

## Introduction

The circulating proteome includes thousands of proteins naturally secreted from cells or present because of cell damage or cell death^1^. These proteins include cytokines, growth factors and hormones and have been shown to be modified by environmental factors and risk pathways such as obesity and conditions such as cancer^2,3^. Protein levels can also predict the risk of certain outcomes^4^, and taken together have an exciting potential to provide biomarkers with potential clinical utility. Exploring protein changes associated with excess adiposity by using causal analyses could help with identifying targets to prevent or reduce adverse health outcomes such as type 2 diabetes (T2D), coronary artery disease (CAD), musculoskeletal diseases and many types of cancer^5–7^. With the widespread availability of proteomic datasets in increasingly large population samples, such investigations have become practically feasible.

Integrating evidence from independent sources which each have their own specific limitations and potential biases (known as triangulation)^8^, can be an important tool in overcoming specific limitations to any one analytical technique/study design. In this context, there are both surgical and non-surgical approaches to weight loss which present opportunities for the examination of the circulating proteome in the context of variation in body mass index (BMI). Clinical trials have implemented interventions in an attempt to induce weight loss; these interventions include bariatric surgery (also referred to as surgical weight loss) and caloric restriction. Bariatric surgery and caloric restriction have been shown to achieve weight loss in people with obesity, with bariatric surgery on average achieving greater weight loss^9^. Previous studies have characterized the effects of individual weight loss interventions on circulating proteins at a relatively small scale^10–19^. Despite these scenarios offering independent records of weight loss, there has not been a comparison of broad proteomic effects of caloric restriction and bariatric surgery, nor either of these in comparison to Mendelian randomisation (MR) results investigating the likely impact of BMI. Consistent evidence across independent trials may help in pointing towards common BMI-driven signals.

In order to study the causal effects of BMI, previous MR studies are useful as these studies have examined the effect of BMI on the plasma proteome using population-based samples^20,21^. MR is a technique that uses a genetic variant or variants to act as proxies for modifiable risk factors of interest, such as BMI, in order to estimate effects of that risk factor on an outcome^22^. Although MR helps to overcome confounding and reverse causation, MR also has limitations. For example, the assumptions of MR^23^ may be violated, especially in instances where traits that are instrumented are complex traits such as BMI. In this case, genetic variants may then not only yield apparent differences in circulating protein levels through variation in BMI, but by other biological processes which are statistically indistinguishable from BMI effects. Further, it is well known that large sample sizes are required for MR^24^, which has only recently become achievable for collections of proteomic data^25,26^.

The aim of this study was to determine the effect of BMI on the proteome. To achieve this aim there were four main objectives of this study. First, use proteomic data from a caloric restriction randomised controlled trial (RCT) to identify the effect this has on circulating proteins. Second, use proteomic data from a bariatric surgery RCT to identify the effect of surgical weight loss on circulating proteins. Third, compare the effects across study designs by identifying proteins with consistent evidence across the surgical and diet-based interventions and incorporating (previously published) BMI to protein abundance MR estimates from a UK-based blood donor cohort (INTERVAL), as a further line of evidence to determine whether protein effects are likely to be driven by BMI. Finally, for proteins that have evidence across the three study designs for a role of BMI in their regulation, utilise existing information about drug targets to characterise the function of proteins in health and disease.

## Results

**Figure 1:**
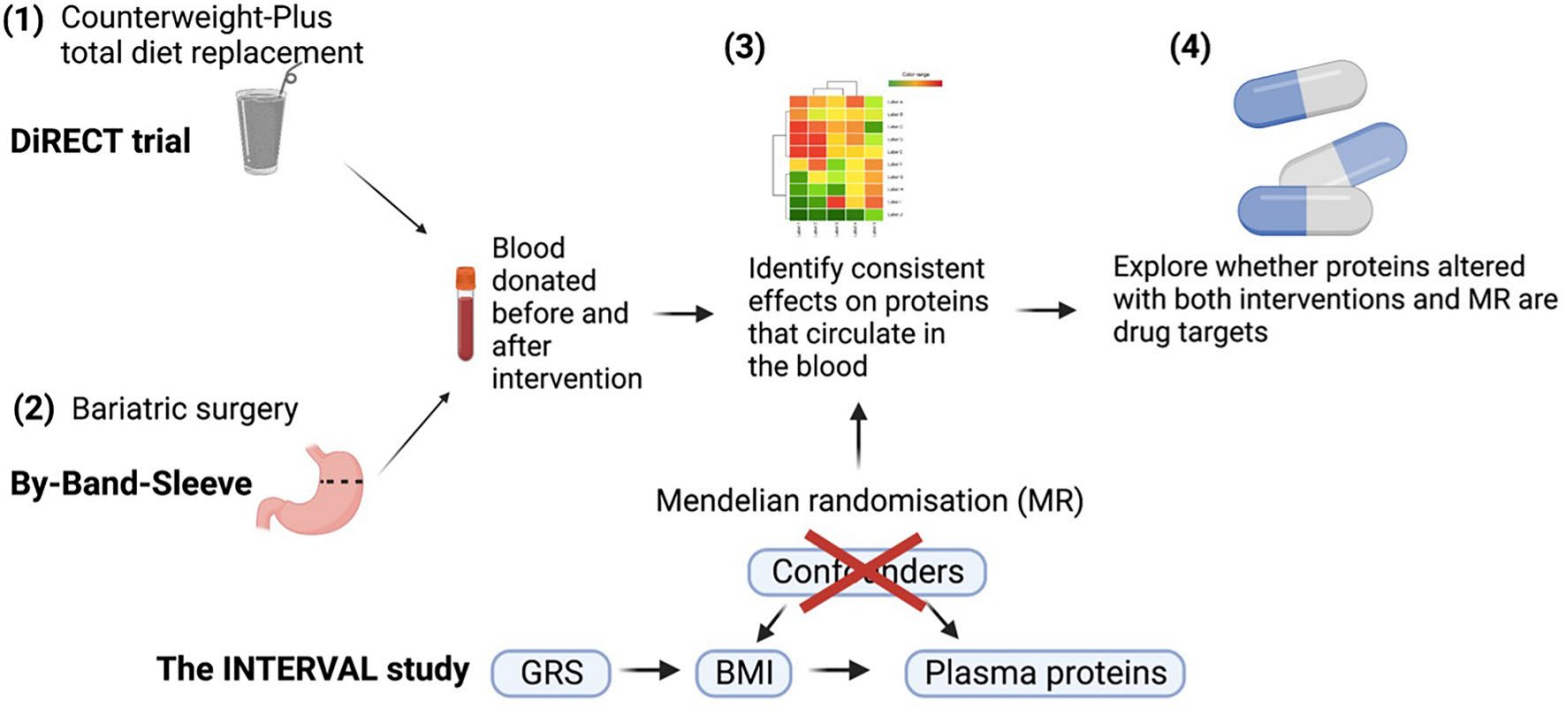
Study overview. GRS = genetic risk score (used as the instrumental variable). BMI = body mass index.

An overview of the results that will be presented is shown in Fig. 1.

### Diabetes Remission Clinical Trial (DiRECT) participant characteristics

Participant characteristics for those included in the caloric restriction trial and in the current proteomic analysis are displayed in Table 1, where N = 292. There were more males than females in the study (55% males in the intervention group and 62% males in the control group) and this is possibly a reflection of the patient population more generally^27^. Participants were older and had higher high-density lipoprotein (HDL)-cholesterol in the control group than those in the intervention group. However, other characteristics were similar across treatment and control groups. Baseline BMI in the control group and intervention group was 35.1 kg/m^2^ (SD 4.6 kg/m^2^) and 34.3 kg/m^2^ (SD 4.3 kg/m^2^) respectively. All participants had a T2D diagnosis (mean 3 years, SD 1.8 years) and the majority were on medication for control of their diabetes. Mean HbA1c levels in the control group were 60 mmol/mol (SD 14 mmol/mol) and 58 mmol/mol (SD 12 mmol/mol) in the intervention group, while the target HbA1c level for a patient with T2D is < 48 mmol/mol^28^. The participants in the control group had a mean reduction in BMI of 0.34 kg/m^2^ (SD 1.3 kg/m^2^) and the intervention group had a mean reduction in BMI of 3.50 kg/m^2^ (SD 2.8 kg/m^2^). Characteristics were mostly similar across males and females (Supplementary Table 1), except females had higher levels of total cholesterol and HDL-cholesterol and a higher BMI (35.3 kg/m^2^, SD 4.6 kg/m^2^ in females vs 34.3 kg/m^2^ (SD 4.2 kg/m^2^) in males, p = 0.04). The cohort is representative of diabetes patterns in the wider community as women tend to have a higher BMI than men to develop T2D^29^.

**Table 1:** Baseline characteristics of the DiRECT trial participants who underwent the intervention (total diet replacement).

Baseline variable		Intervention group (mean ± SD or n (%))	Intervention group N	Control group (mean ± SD or n (%))	Control group N	P-value for difference
Age		53.0 (7.5)	146	56.2 (7.1)	146	2.3 × 10^–4^
Sex	Male	55%	146	62%	146	0.28
	Female	45%		38%		
BMI (kg/m^2^)		35.1 (4.6)	146	34.3 (4.3)	146	0.10
Systolic blood pressure (mmHg)		133 (17)	146	137 (16)	146	0.03
HbA1c (mmol/mol)		60 (14)	146	58 (12)	146	0.13
Glucose (mmol/l)		9.2 (3.3)	146	8.8 (2.6)	144	0.23
Insulin (mIU/ml)		25 (15)	146	23 (14)	144	0.26
Cholesterol (mmol/l)		4.3 (1.2)	146	4.3 (1.2)	143	0.91
HDL (mmol/l)		1.1 (0.3)	146	1.2 (0.3)	143	9.5 × 10^–3^
Triglycerides (mmol/l)		2.1 (1.4)	146	1.9 (0.9)	143	0.34
Diabetes duration (y)		3.0 (1.7)	146	3.0 (1.8)	146	0.81
Number of anti-diabetic medications		1.14 (0.94)	146	1.09 (0.83)	146	0.60
Centre	Scotland	78%	146	57%	146	1.8 × 10^–4^
	Tyneside	22%		43%		
List size	> 5700	62%	146	50%	146	0.06
	≤5700	38%		50%		

Sample size (N) is indicated and is up to 146 where there is no missing data. A two-tailed unpaired student’s t-test was used to compare continuous variables and a Chi-squared test was used to compare differences in categorical variables.

### DiRECT caloric restriction effect on circulating protein levels

**Figure 2:**
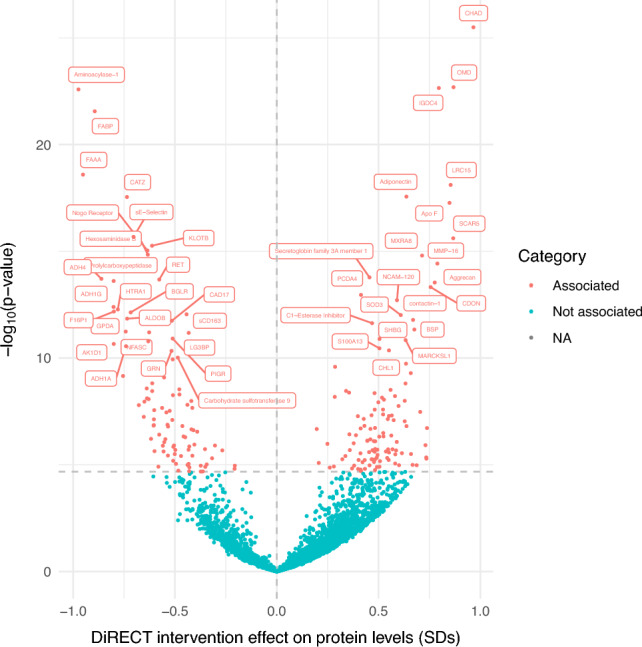
Volcano plot displaying the linear mixed model results in DiRECT. Proteins were categorised as associated if p < 1.2 × 10^–5^. “Associated” proteins are labelled where labels are non-overlapping. Full protein names can be found in Supplementary Table 2.

A linear mixed model was used to estimate the effect of the caloric restriction intervention on proteins, providing an estimate of the change in protein levels in normalised SD units. There was evidence that caloric restriction, which was associated with a reduction in weight/BMI, affected circulating levels of 216 out of 4601 proteins (5%) at a conservative Bonferroni adjusted p < 1.2 × 10^–5^ (Supplementary Table 2). Of these, levels of 120 proteins increased after the intervention and levels of 96 proteins decreased (Fig. 2). Proteins that had the strongest evidence for an increase following the intervention include chondroadherin (CHAD, beta = 0.97 normalised SDs, 95% CI 0.80 to 1.13, p = 3.2 × 10^–26^), osteomodulin (OMD, beta = 0.87 SDs, 95% CI 0.71 to 1.02, p = 2.05 × 10^–23^) and Immunoglobulin superfamily DCC subclass member 4 (IGDC4, beta = 0.80 SDs, 95% CI 0.65 to 0.94, p = 2.4 × 10^–23^). Proteins most strongly decreased include aminoacyclase-1 (beta = −0.97 SDs, 95% CI −1.15 to −0.80, p = 2.6 × 10^–23^), fatty acid-binding protein (heart) (FABP3, beta = −0.89 SDs, 95% CI −1.06 to −0.73, p = 2.8 × 10^–22^) and fumarylacetoacetase (FAAA, beta = −0.95 SDs, 95% CI −1.14 to −0.76, p = 2.6 × 10^–19^). Full results are available in Supplementary Table 2. In some cases, proteins that have previously been reported as BMI-associated had betas that were directionally consistent with reported effects but did not pass our multiple testing threshold for association, for example CRP (beta = −0.42 SDs, 95% CI −0.64 to −0.22, p = 7.9 × 10^–5^).

### By-Band-Sleeve characteristics at baseline

A subset of participants (N = 125) from a bariatric surgery trial (By-Band-Sleeve) were included in the proteomics study. Participants had study visits at baseline (randomisation) and ~ 36 months post-randomisation (median = 33 months post-surgery, range = 21 to 44 months). Most participants in the By-Band-Sleeve proteomics study were female (69%) (Table 2). They had a mean age of 50 years (SD 10 years) and a mean BMI at baseline of 45 kg/m^2^ (SD 8 kg/m^2^). Age and BMI were similar in males and females. Forty-three participants (36%) had been diagnosed with T2D with a mean duration of 6 years and 4 months (SD 5 years 5 months). There was evidence for some sex differences across participant characteristics: males had higher rates of T2D (61%. vs 26% females, p < 2.2 × 10^–4^). There was also a difference between cardiometabolic traits, where females had higher levels of low density lipoprotein (LDL)-cholesterol, HDL-cholesterol and total cholesterol, whereas males had higher levels of triglycerides. As the trial is ongoing at the time of writing, the exact reduction in BMI cannot be reported. Based on existing literature, the expected reduction in BMI across bariatric surgery subtypes is around 10 kg/m^2^^30^. Published results by the By-Band-Sleeve Trial Management Group and Investigators provides further baseline information on all participants in the By-Band-Sleeve trial (not just the subset whose samples were sent for proteomic analysis)^31^.

**Table 2:** Characteristics of participants included in the analysis for the By-Band-Sleeve trial. Sample size (N) is up to 118 where there was no missing data.

Characteristic	All participants (mean ± SD or n (%))	N	Male, N = 36	Female, N = 82	p-value
Age (years)	50 (10)	118	50 (9)	50 (10)	0.76
Body mass index (BMI) (kg/m^2^)	45 (8)	118	46 (7)	45 (8)	0.27
Baseline systolic blood pressure (mmHg)	136 (15)	118	138 (15)	136 (15)	0.70
Baseline diastolic blood pressure (mmHg)	84 (7)	118	85 (8)	83 (7)	0.30
Smoking category		118			0.069
Never	49 (42%)		14 (39%)	35 (43%)	
Ex-smoker	64 (54%)		18 (50%)	46 (56%)	
Rarely or occasionally	5 (4.2%)		4 (11.1%)	1 (1.2%)	
Diabetic (diet, oral treated or insulin)	42 (36%)	118	22 (61%)	21 (26%)	2.2 × 10^–4^
Time since diabetes diagnosis (months)	76 (65)	118	72 (49)	80 (79)	0.63
Ethnicity		118			0.70
English/Welsh/Northern Irish/Scottish/British	110 (93%)		33 (92%)	77 (94%)	
Other (Any other white background, Irish, Gypsy/Traveller, White and Black Caribbean)	8 (7%)		3 (8%)	5 (6.1%)	
Employment status		118			8.3 × 10^–3^
Full-time	38 (32%)		16 (44%)	22 (27%)	
Part-time	27 (23%)		5 (14%)	22 (27%)	
Self-employed	9 (7.6%)		6 (17%)	3 (3.7%)	
Other (Homemaker/Student/Retired/Unable to work/Unemployed)	44 (37.2%)		9 (25%)	35 (43%)	
Income		114			0.91
< £10,000–20,000	53 (46%)		18 (51%)	35 (44%)	
£20,001-£40,000	38 (33%)		11 (31%)	27 (34%)	
> £40,001	23 (20%)		6 (17%)	17 (22%)	
Baseline triglycerides (mmol/L)	1.59 (0.76)	118	1.83 (0.74)	1.48 (0.75)	3.6 × 10^–3^
Baseline LDL-cholesterol (mmol/L)	2.71 (0.96)	117	2.39 (0.89)	2.86 (0.96)	0.014
Baseline HDL-cholesterol (mmol/L)	1.21 (0.27)	118	1.03 (0.17)	1.29 (0.27)	8.7 × 10^–7^
Baseline total cholesterol (mmol/L)	4.65 (1.10)	118	4.25 (0.94)	4.83 (1.12)	9.3 × 10^–3^

Categorical variables were compared using a Chi-squared test and continuous variables were compared using a Wilcoxon signed rank test.

### Bariatric surgery effect on protein levels

**Figure 3:**
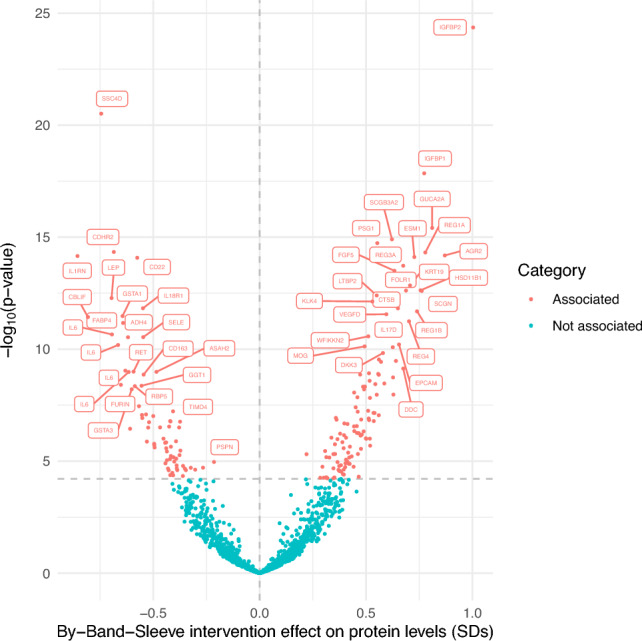
Volcano plot of the change in protein levels after bariatric surgery using a linear mixed model. Proteins were categorised as associated if p < 6.2 × 10^–5^. Full protein names and effect estimates can be found in Supplementary Table 3.

A linear mixed model was implemented to estimate the effect of bariatric surgery on protein levels, providing an estimate of the change in protein (in normalised SD units) following the intervention. The serum levels of 191 proteins out of 1472 (13%) were associated with the bariatric surgery intervention (p < 6.2 × 10^–5^) in the By-Band-Sleeve study, with 118 proteins showing higher levels and 73 proteins showing lower levels post-surgery (Fig. 3, Supplementary Table 3). Of all proteins associated with the intervention, the maximum percentage of samples below the specified limit of detection (LOD) was 28% (mean = 1.0%, range = 0-28%). The protein most strongly increased after bariatric surgery was insulin-like growth factor binding protein 2 (IGFBP2, beta = 1.00 SDs, 95% CI = 0.85 to 1.15, p = 4.3 × 10^–25^). Other proteins that increased following bariatric surgery include insulin-like growth factor binding protein 1 (IGFBP1, beta = 0.77 SDs, 95% CI 0.63 to 0.92, p = 1.4 × 10^–18^), guanylin (GUCA2A), pregnancy-specific beta-1 glycoprotein 1 (PSG1) and ficolin-2 (FCN2, beta = 0.69 SDs, 95% CI 0.52 to 0.85, p = 2.4 × 10^–13^). Bariatric surgery reduced the levels of other proteins, such as the scavenger receptor cysteine rich domain-containing group B protein (SSC4D, beta = −0.74 SDs, 95% CI −0.87 to −0.62, p = 3.1 × 10^–21^), cadherin-related family member 2 (CDHR2) and B-cell receptor CD22. Proteins with an established relationship with BMI such as leptin^20^ were reduced with the bariatric surgery intervention (beta = −0.70 SDs, 95% CI −0.86 to −0.53, p = 5.2 × 10^–13^). Full results are provided in Supplementary Table 3.

### Comparison of intervention effects on protein levels

**Figure 4:**
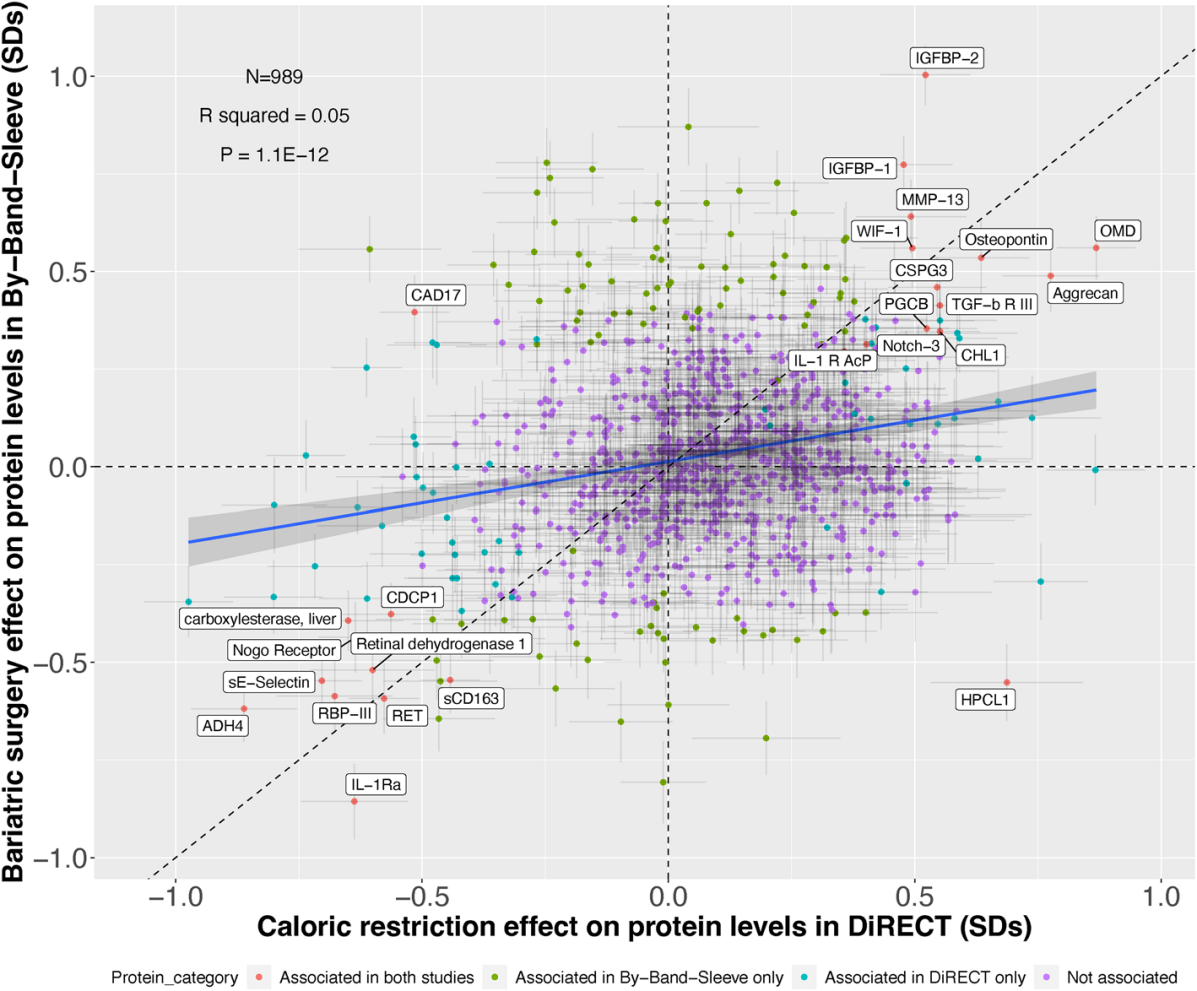
Comparison of estimates for the change in protein levels following either the total diet replacement in DiRECT or bariatric surgery in By-Band-Sleeve. Proteins denoted as "associated" passed the pre-defined adjusted p-value thresholds in one or both analyses. Effect estimates are the change in protein levels in normalised SD units comparing endpoint (post intervention) levels to baseline. The diagonal dashed line indicates y = x and the blue solid line is the regression line with 95% confidence intervals . Proteins that were associated in both studies are labelled.

There were 989 unique proteins matched by UniProt ID when combining the 4601 protein measurements from the DiRECT trial and 1472 protein measurements from the By-Band-Sleeve trial. Supplementary Table 4 provides a summary of the effect estimates for shared proteins. Among the 989 proteins in the merged data set, 81 proteins (39 with a positive direction, and 42 with a negative direction) were associated with caloric restriction and 130 proteins (84 with a positive direction and 46 with a negative direction) were associated with bariatric surgery. A total of 25 proteins were detectably associated with both interventions: 23 of these were consistent in direction of change and two had opposite effects. 56 of the 81 proteins associated only with caloric restriction, and 105 of the 130 proteins associated only with bariatric surgery (Fig. 4). The proteins with the strongest effects and that had consistent directions of effect include intervention-increasing effects on IGFBP1/2, osteomodulin (OMD), and intervention-lowering effects on pro-inflammatory proteins such as interleukin-1 receptor antagonist protein (IL-1Ra), scavenger receptor cysteine-rich type 1 protein M130 (sCD163) and E-selectin (sE-selectin). Proteins were measured in the two studies using different proteomic technologies, platforms run by SomaLogic (in DiRECT) and Olink (in By-Band-Sleeve). Among the 23 proteins with consistent effects across interventions, 19 feature in a recent analysis comparing quantification of proteins by these platforms; of these, all 19 were found to be positively correlated across platforms^32^ (mean r = 0.71, range = 0.35 to 0.96).

Two of the proteins associated with both interventions had opposite directions of effect. The CAD17 protein was found to be increased by bariatric surgery but decreased by caloric restriction, and the HPCL1 protein was reduced by bariatric surgery but elevated by caloric restriction (Fig. 4). Correlation data between SomaLogic and Olink platforms for these two proteins with discordant effects was not available from Haslam et al*.*^32^ since the Olink panel used in that study had fewer unique proteins (N = 972)^32^ than the ones measured in this study.

### Comparing intervention effects on protein levels with MR estimates

**Figure 5:**
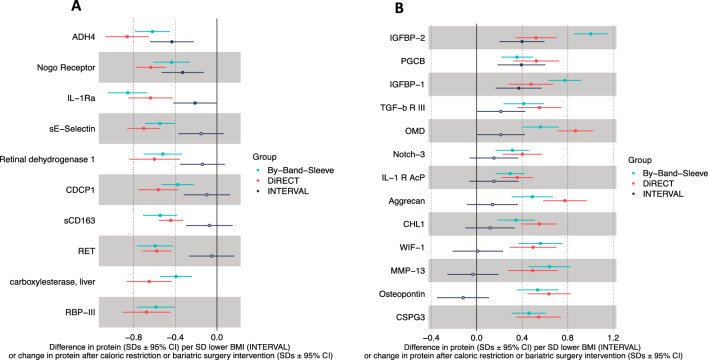
Forest plots comparing the estimates for the effect of interventions on protein levels (based on a linear mixed model) with published MR results from the INTERVAL study (where beta coefficients represent an estimate of the mean protein change per SD lower BMI). The MR estimates from Supplementary Table 5 have been multiplied by −1 so that the units are the difference in protein per SD lower BMI. (**A**) Proteins that were reduced with total diet replacement (TDR) and bariatric surgery. (**B**) Proteins that were increased following TDR and bariatric surgery.

Previously published one-sample MR results provided estimates of the difference in protein levels per 1 SD (~ 4.8 kg/m^2^) higher BMI^20^. These estimates are provided in Supplementary Table 5. The estimates for the effect of each intervention on proteins were compared with one sample BMI to protein MR estimates to identify whether intervention effects are consistent with the BMI-associated causal effect estimates^20^. To provide an estimate to match the intervention effects, the MR estimates were multipled by −1 to indicate the mean difference in protein in normalised SDs per normalised SD lower BMI. For example, if the MR estimate suggests that lower BMI results in a lower level of a protein, we would predict that the protein would be reduced with the interventions. Out of the 23 proteins which displayed consistent effects with both interventions, 20 proteins had available MR estimates. As the underlying populations have different BMI distributions, and the trials likely induce a different degree of weight loss, the estimates presented only allow for interpretation of direction effect, rather than direct comparison of the magnitudes of effect. For all but two proteins, the direction of effect of the BMI to protein MR estimate was consistent in direction with the intervention effect estimates (Fig. 5A,B). Six of these MR estimates had 95% confidence intervals that did not overlap the null, providing a short list of consistently associated proteins across all three studies (DiRECT, By-Band-Sleeve, and INTERVAL) (Table 3).

**Table 3:** Exploration of proteins modified by weight loss interventions and in a Mendelian randomisation framework as therapeutic targets.

Gene	Target	Protein full name	Direction of relationship with BMI	Current or potential to be small-molecule target^1^	Current or potential to be biologic target^1^	Protein involved in ADME^1^	Tier^1^	Drug which involves protein target and therapeutic use (approved only)^2^
*IGFBP1*	IGFBP1	Insulin-like growth factor binding protein 1	Lower BMI increases protein	Y	Y	N	2	**Mecasermin:** IGFBP1 is a carrier for this recombinant IGF1. Used in growth failure as a result of insulin-like growth factor 1 deficiency in paediatric patients
*ADH4*	ADH4	Alcohol dehydrogenase 4	Lower BMI reduces protein	Y	N	Y	1	**Ethanol**: chronic pain **NADH:** nutritional supplement
*RTN4R*	Nogo Receptor	Reticulon-4 receptor	Lower BMI reduces protein	Y	Y	N	3A	NA
*IL1RN*	IL-1Ra	Interleukin-1 receptor antagonist protein	Lower BMI reduces protein	N	Y	N	3A	NA
*IGFBP2*	IGFBP2	Insulin-like growth factor binding protein-2	Lower BMI increases protein	Y	Y	N	2	**Mecasermin:** IGFBP2 is a carrier for this recombinant IGF1. Used in growth failure as a result of insulin-like growth factor 1 deficiency in paediatric patients
*BCAN*	PGCB	Brevican core protein	Lower BMI increases protein	N	Y	N	3B	NA

^1^Information from Supplementary Table 1 in Finan et al.^34^ Y = yes, N = no. Small molecule = chemically derived, biologic = extracted from living organism. ^2^Information from DrugBank^33^. ADME = absorption, digestion, metabolism, excretion^34^. Tier 1 = target with approved small molecule or biologics, or drug candidate in clinical phase. Tier 2 = target with known bioactive drug-like small molecular binding partners or have high similarity to approved drug targets. Tier 3 = structural similarities to approved drugs (to a lesser degree than Tier 2) or are a protein with known potential to be targeted (such as a G protein-coupled receptor)^34^. More details can be found by Finan et al.

The two proteins, CAD17 and HPCL1, that were associated with both interventions but in opposite directions did not have published MR estimates available. Furthermore, it is unclear whether these seemingly opposing effects represent meaningful heterogeneity across interventions and/or study samples or if the discordance of effect is a result of differences in the way these proteins are measured by the two platforms (no correlation statistics are available from Haslam et al*.*^32^ in this case).

### Are proteins altered by body composition druggable targets?

For proteins with evidence of consistent effects across both interventions and in an MR framework, we explored whether the proteins are current drug targets to help understand the relevance of the proteins to comorbidities of BMI. To do this, we searched for the relevant targets using DrugBank^33^ and searched protein targets identified by Finan et al^34^. IGFBP1 and IGFBP2 have approved drugs to treat disorders such as growth failure^33^. ADH4 is a target for nutritional supplementation^33^. Other proteins suggested as having the potential to be drug targets include RTN4R^34^, IL1-Ra^34^ and brevican core protein (BCAN/PGCB)^34^. These do not currently have approved therapeutic interventions, but based on their structure they may have potential to be a drug target. A summary of these targets and the currently approved drugs is provided in Table 3.

## Discussion

This study provides an in-depth characterization of the effect of weight loss on circulating proteins by combining the results from two weight loss interventions that are routinely used by health services: a low-calorie TDR and bariatric surgery. Results demonstrated that the low-calorie diet and bariatric surgery had a broad effect on protein levels. These analyses identified consistent evidence for an effect of weight loss interventions on 23 proteins. Integrating results from a recently published MR study estimating the causal effect of BMI on plasma proteins provided a third line of evidence for a causal role of BMI in the circulating levels of 6 of the 23 proteins, with estimates that were consistent in direction for 18 out of 20 proteins with available MR estimates. Through exploring these proteins as drug targets, we found that some are currently involved in treating growth failure and others have the potential to be therapeutically targetable if they are found to have a role in disease.

Previous studies have explored the effect on the proteome of either caloric restriction^10–15^ or bariatric surgery^16–19,35,36^ as a means of characterising the proteomic features of BMI. However, this study was able to advance this by comparing proteomic effects across two common weight loss treatments^37^. Our results demonstrated estimates that agreed with effects previously observed by other groups. These changes include a low-calorie diet increasing levels of IGFBP1^11,14^, IGFBP2^12,14^ and interleukin 1 receptor accessory protein (IL1RAP)^10,14^ as well as reducing levels of RTN4R^14^. We also replicated effects observed in bariatric surgery studies, where surgical weight loss led to increased levels of IGFBP1 and IGFBP2 and a reduction in levels of FCN2^18,19^. IGFBP1 and IGFBP2 are known to be involved in the transport of IGF-1 and have a role in glucose metabolism and insulin sensitivity^38,39^. Higher levels of IGFBP-2 have been shown to be associated with higher insulin sensitivity, and lower levels of plasma insulin following bariatric surgery, possibly due to increased IGFBP-2 mRNA expression following surgically induced weight loss^40^. The comparison of the effect estimates with existing studies provides us with confidence that we can capture robust proteomic effects resulting from bariatric surgery and caloric restriction and suggest that a comparison of effects across study designs is appropriate.

The ability to identify consistent proteomic effects across independent evidence sources is one of the key strengths of the study. Bariatric surgery and caloric restriction resulted in a reduction of proteins associated with inflammation such as IL-1Ra^41^, sCD163 and sE-selectin. sCD163 and sE-selectin are soluble receptors: sCD163 is shed from macrophages/monocytes under inflammatory conditions^42^ and sE-selectin is involved in the adhesion of leukocytes to the endothelial wall and plays a role in atherosclerosis^43^. Both interventions altered circulating levels of ADH4, which was supported by MR analyses. ADH4 is a protein in the alcohol dehydrogenase family, which catalyses NAD-dependent oxidation and is important in alcohol metabolism. Circulating levels of ADH4 have been shown to be associated with BMI observationally^25^, however evidence is limited on whether levels of this protein are altered with a weight loss intervention. MR studies have provided evidence that non-alcoholic fatty liver disease (NAFLD) raises levels of ADH4^44^, therefore weight loss reducing ADH4 could be an indicator of an improvement in liver function. Levels of ADH4 were also positively associated with the polygenic risk score for incident T2D^45^, therefore it is possible that ADH4 could be involved in the relationship between adiposity and T2D. This study also found consistent effects across the three study designs that lower BMI raises levels of BCAN. The gene encoding brevican core protein (*BCAN)* is highly expressed in the central nervous system and is thought to have a role in the formation of the brain extracellular matrix^46^. The levels of BCAN were shown to be increased 12 years following bariatric surgery^19^; our study supports that the effect can also be seen after a shorter bariatric surgery follow up of three years, and 1 year following a dietary intervention. A recent study suggested that higher levels of BCAN may be linked to a reduction in cardiovascular-specific mortality^47^.

One way to explore the function of proteins altered by BMI is to look at whether drugs which target these proteins have an approved therapeutic use. Proteins such as IGFBP1 and IGFBP2 are currently targeted by drugs to treat growth disorders^33^. Other proteins altered by BMI and weight change have been suggested to be potential drug targets, but currently do not have any approved drugs acting at the protein, such as the RTN4R, IL-1Ra and BCAN^34^. Although no approved drugs, an antagonist for RTN4R was in clinical trials for the treatment of multiple sclerosis (however no development has been reported in such trials). Drugs which mimic IL-1Ra are currently in Phase II clinical trials for the treatment of urinary tract infections (Citeline Pharma Intelligence citeline.informa.com). If these proteins are established as having a role in disease, they could be useful targets for future drug repurposing/development. Previous studies comparing protein levels in individuals with and without T2D^48^ suggests that the changes in levels of proteins, including ADH4, RTN4R, IGFBP2 and BCAN, following the weight loss interventions support a proteomic signature of remission of T2D. Further characterization of the role of these proteins in physiological processes and in disease is required through use of MR and laboratory studies. Despite the possibility that changes in protein composition related to changing BMI may have adverse downstream effects, it is also important to recognise that changes in some (or many) of these proteins may simply be biomarkers for adiposity. The exploration of these proteins as drug targets is just one of many tools that should be employed to understand how a change in circulating proteins relates to health.

Although we have data from two comprehensive and well validated technologies, and are making good use of existing data, the two trials included in the current study utilized different proteomic technologies. Where possible, we explored the correlation of proteins across Olink and the SomaScan® by incorporating published correlation coefficients and have provided these results in full. Here, a large proportion of proteins which had evidence for consistent effects with both interventions had strong correlations. We therefore believe that the utility of comparing estimates across studies outweighs the limitations that arise by cross-platform comparisons. Second, it is important to note that proteomic profiling was run on plasma samples in the DiRECT study and in serum in By-Band-Sleeve. Serum is the liquid remaining once the blood has been allowed to clot, whereas plasma is derived from blood that has not clotted as it has been taken into a vacutainer containing an anticoagulant^49^. Proteins may have different absolute abundance in plasma or serum, however the ability to detect changes in protein levels should be similar^50^. The two trials had differing follow-up times, with By-Band-Sleeve at 3-years and DiRECT at 1-year. All participants in DiRECT had T2D, whereas By-Band-Sleeve had a mix of participants with and without T2D. By-Band-Sleeve also had a higher proportion of females. Bariatric surgery and caloric restriction generally induce differing degrees of weight loss, therefore the absolute magnitude of the changes in proteins across interventions are not directly comparable. Whilst these differences in patient populations could explain some of the discordance in protein effects across interventions, the heterogeneity across studies is a strength where observing consistent protein effects. Analyses performed in the current study estimated the average effects across both males and females, however, future work could include exploring sex-specific effects within these studies.

Overall, this study has provided an extensive characterisation of caloric restriction or bariatric surgery interventions at the level of circulating proteins. We have exemplified how triangulation, using two independent trials and an MR study, can be used to identify proteins that are affected by body composition and which may play a role in the considerable pathophysiology associated with excess adiposity. Further MR and laboratory studies are required to investigate the role of these proteins in health and disease.

## Methods

### Datasets and statistical analysis

#### DiRECT study overview

Samples analysed were collected from participants enrolled in the DiRECT trial. Participants enrolled were between 20 and 65 years of age, diagnosed with T2D within the previous six years and had a BMI of between 27 and 45 kg/m^2^. Ethics approval was granted by West 3 Ethics Committee in January, 2014, with approvals by the National Health Service (NHS) health board areas in Scotland and clinical commissioning groups in Tyneside^51^. A statistical analysis plan was written and access to data was granted by DiRECT trial prinicipal investigators. All methods were performed in accordance with the relevant guidelines and regulations. Participants were excluded if: they were using insulin, had a glycosylated haemoglobin (HbA1c) concentration of ≥ 12% (≥ 108 mmol/mol), had more than 5 kg weight loss in the preceding six months and/or had an estimated glomerular filtration rate of < 30 mL/min per 1.732 m^2^. Other exclusion criteria include malignancy, heart failure, recent myocardial infarction (< 6 months), enrolment in other clinical trials, addiction to illegal drugs, difficulty in understanding the study, current use of drugs to treat obesity, eating disorders, pregnancy or admission to hospital for depression or use of antipsychotic medication^51^. General practitioner (GP) practices were assigned to control or intervention, which was dependent on the practice list size (number of patients registered to each practice). This was done to ensure that the intervention/control allocations were balanced across centres and list size (small ≤ 5700 or large > 5700). Therefore, centre and list size are variables used for stratified randomization^27^. Participants in the control group received best-practice care by guidelines. The intervention group followed the Counterweight-Plus weight management programme^52^. This programme involved a total diet replacement (TDR) phase using a low energy diet (825–853 kcal/day) for 3–5 months. Following the TDR there was a structured food reintroduction phase of 2–8 weeks. Participants then attended monthly weight loss maintenance visits. Those in the intervention group had their antidiabetic and antihypertensive drugs discontinued. In total, there were 306 individuals recruited into the study, with 149 patients included in each intention-to-treat population (in both intervention and control groups) after removal of participants that had been randomised in error or removed consent^51^.

Age and sex were self-reported. Height was measured with the Frankfort plane horizontal, with a portable stadiometer (Chasmors Ltd, London). Weight was measured using Class 111 approved calibrated scales (Marsden Group UK). Blood was donated at various timepoints including at baseline (week 0) and at 1 year (~ week 52), when HDL-cholesterol, triglycerides, HbA1c and plasma glucose were measured. Systolic blood pressure was measured with the patient seated, rested and with legs uncrossed for ≥ 5 min. BMI (kg/m^2^) was calculated by dividing the weight (kg) by the square of the height (m).

#### DiRECT proteomics and statistical analysis

**Figure 6:**
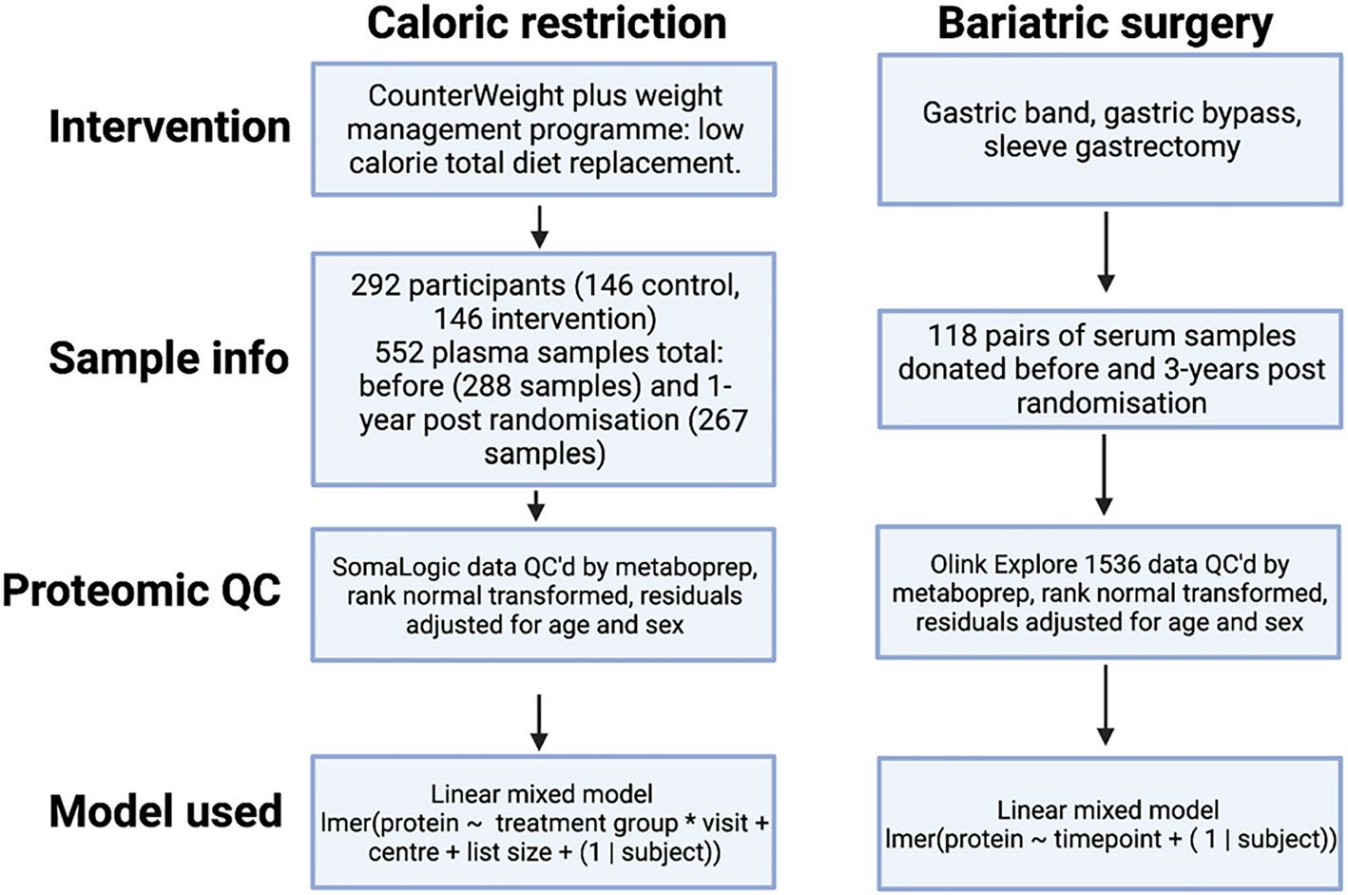
Overview of the two interventions used and a summary of analyses performed.

Blood was taken from participants by venipuncture into 9 mL vacutainers with ethylenediaminetetraacetic acid (EDTA) at baseline and at 1-year post-randomisation. Blood samples were centrifuged to derive plasma samples and plasma was stored at − 80 °C. Protein detection was performed by the SomaScan® assay by SomaLogic. This was performed on 569 samples from 302 individuals. This technique uses Slow Off-rate Modified Aptamers (SOMAmers) which make direct contact with proteins and quantifies protein levels in relative fluorescence units (RFUs) by using a DNA microarray^53^. This quantification is a product of both the affinity of the SOMAmer for the target and the concentration of the protein. Measurements returned by SomaLogic had undergone internal processing, where data were hybridised control normalised, intraplate median signal normalised, plate scaled, calibrated and adaptive normalized; further details of these adjustments can be found in the technical note by SomaLogic^54^. There were 5284 proteins included in the array, of which 4601 proteins remained after internal technical quality control (QC), including the removal of non-human proteins. The proteomic data were then subject to a study-level QC using the “metaboprep” R package^55^, with data from both timepoints QC’d together. Although this package was primarily developed for use with metabolomic data, the functions are also applicable to proteomic data. The following input parameters were used for exclusion of protein measurements: extreme missingness (> 80%) for each individual or each protein, user defined missingness of > 20% for each individual or each protein, protein measurement > 5 SDs from the total peak area (sum of protein level for each individual at proteins with no missingness), and > 5 SDs from the mean of principal complements (PCs) PC1 and PC2. These filtering criteria excluded 4 samples based on PC outliers, leaving 565 samples from 300 participants. On merging with clinical data (as analysed in the primary results paper^51^), 292 participants (146 per study group) and 552 samples remained and were therefore included in statistical analyses. In the control group, there were 145 samples at baseline and 143 at endpoint. In the intervention group, there were 143 samples at baseline and 121 samples at endpoint (Fig. 6).

The metaboprep package also calculated the number of independent proteins by using pairwise Spearman’s correlation coefficients between proteins (2380 representative proteins based on correlation coefficient of 0.5). The Shapiro–Wilk test was implemented to identify proteins which have a normal distribution (W ≥ 0.95). Only 644 out of 4601 proteins had W statistics ≥ 0.95 and therefore all data were transformed to meet normality assumptions for analyses. Data were rank-based inverse normal transformed to give a mean of 0 and a standard deviation of 1 and data were adjusted for age and sex. The units of protein measurements are therefore in normalised SD units. Analyses were performed using R version 3.6.1.

The effect of the intervention on plasma proteins was estimated using a linear mixed model (lmer() function from the “lme4” R package). Within this model, the timepoint (visit), treatment group, centre and list size were included as fixed effects and the subject was included as a random effect. The centre and list size of each GP practice were stratification variables within the trial, therefore they were included as covariables^56^. The estimate for the effect of the intervention on protein was reported as the interaction coefficient for treatment group (with control group as reference) and timepoint (with baseline as reference), where the direction of effect indicates the change in protein level when comparing endpoint to baseline (i.e. a negative slope indicates the intervention reduces the level of the protein). A p-value was derived by performing an ANOVA of two fitted models, one including and one excluding the interaction term, under the conditions of a maximum likelihood (ML) model. A summary of this analysis is provided in Fig. 6. We used a Bonferroni multiple adjusted p-value of 0.05/2380 = 2.1 × 10^–5^ to guide strength of associations based on the number of representative proteins at a Spearman’s correlation coefficient of rho = 0.5. All effect estimates, measures of precision and p-values are presented in full in the supplementary material.

#### By-Band-Sleeve study overview

By-Band-Sleeve is a multi-centre trial which aims to determine which bariatric surgery type is the most effective for weight loss and quality of life (out of Roux-en-Y gastric bypass, the laparoscopic adjustable gastric band and the sleeve gastrectomy) (NIHR09/127/53,UK) at three years after randomisation. The trial was granted research ethics approval by the Southwest Frenchay Research Ethics Committee (reference 11/SW/0248). Written informed consent was obtained from all participants. The study is funded by the NIHR and aims to report this year. This trial began recruitment (as part of an internal pilot phase) in December 2012 in two centres, where the initial bariatric surgeries included the gastric bypass and gastric band^57^. The sleeve gastrectomy was later added as a third bariatric surgery within the trial and the number of participating centres increased to twelve^58^. The trial completed recruitment in September 2019 with 1341 patients having been randomised. The trial reported the cardiovascular disease history, medication, full blood count measurements and cardiometabolic risk factors of participants. Due to the results of the trial not being published, the exact BMI change that occurred in By-Band-Sleeve cannot yet be reported. A statistical analysis plan was written and access to data was granted by By-Band-Sleeve trial prinicipal investigators. All methods were performed in accordance with the relevant guidelines and regulations.

Ten of the twelve participating centres elected to collect samples for future research with all participants enrolled at these centres being given the option to consent to sample collection at baseline (pre-randomisation) and 36-months post-randomization for this purpose. Samples were collected using 4 ml clot activator gel vacutainers and centrifuged at the specific site then stored at − 80 °C. Samples were shipped on dry ice. A subset of these samples were used in this study for proteomic profiling. Specifically, only sample pairs (those collected from the same patient before and after surgery) were selected for proteomics analysis. These sample pairs were collected at Musgrove Park Hospital (Taunton, UK) and were available for analysis as of December 2020.

#### By-Band-Sleeve proteomics and statistical analysis

Samples were thawed and randomly aliquoted across three plates, ensuring pairs of samples (from the same individual) were on the same plate, and sent to Olink in February 2021. This resulted in 250 serum samples from 125 participants being analysed. Within this manuscript, data from the By-Band-Sleeve trial refers to this subset of patients and their data from the trial. Samples were analysed by the Olink Explore 1536 panel (Olink Proteomics, Uppsala, Sweden). Olink uses proximity extension assay (PEA) technology to detect and quantify protein levels^59^. This technology uses pairs of antibodies bound to DNA tags. When the antibodies bind to the protein, the DNA tags hybridise and can be quantified using next generation sequencing Illumina® NovaSeq platform. Proteins are measured in normalized expression (NPX) units which are on a log_2_ scale. This panel provides a maximal readout of 1536 proteins (Supplementary Table 6). Olink detected and returned data for 1472 proteins after excluding proteins that failed internal technical QC procedures. Samples with QC warnings from Olink (internal control deviation of more than ± 0.3 NPX, at least 500 matched counts or deviation of negative controls < 5 SDs of the predefined value) were left in but further study-level QC was implemented using “metaboprep”^55^. Olink flagged proteins which fell below a lower LOD, however as the data were further QC’d by “metaboprep” and subsequently rank-based inverse normal transformed, no proteins were excluded based on the LOD. It has also been reported that including values that are lower than the LOD helps with increasing statistical power and in increasing the normality of the data^50^. Information about the percentage of samples that fell below the lower LOD for each protein are provided alongside the results.

The input parameters for exclusion in “metaboprep” were the same as for the proteomic data in DiRECT. From a total of 250 samples (125 pairs) and 1472 proteins (following Olink QC above), two samples were excluded as PC outliers (leaving 123 complete pairs). As in DiRECT, a Spearman’s rho of 0.5 (tree cut height of 1-rho = 0.5) was selected to determine the number of independent or representative proteins, which totalled 805. The proteomic data were rank-based inverse normal transformed, residuals then adjusted for age and sex, and the residuals used for the main analysis. We removed participants from the analysis if they did not have a surgery date or if the surgery date was after their 36-month post-randomisation appointment, therefore suggesting they had not yet undergone surgery (N = 4). Participants were also removed from analyses if, after study-level sample QC, they had missing proteomic data at one of the timepoints (N = 1). This left 118 individuals for the main analyses.

Similar to DiRECT, a linear mixed model was used to assess the effect of timepoint (at baseline and 3 years after randomisation) on plasma proteins, where the timepoint was added as a fixed effects predictor and subject as a random effects predictor. P-values were derived by comparing models with and without timepoint using an ANOVA, where a multiple testing adjusted p-value of 0.05/805 (number of independent proteins at a correlation of r = 0.5) = 6.2 × 10^–5^ was used to guide strength of associations. R version 4.0.3 was used for the analysis of By-Band-Sleeve data.

#### INTERVAL study

INTERVAL was a trial that aimed to assess the safety and efficacy of reducing the time between blood donations in a population free from major (self-reported) disease. The study enrolled around 50,000 participants. A subsample of the cohort (N = 3,301) also had plasma protein measurements on the SomaScan (SomaLogic) platform. We utilized these MR results which we have previously published to identify proteins with causal evidence for BMI-driven effects^20^. Within the analysis, a genetic risk score (GRS) for BMI was constructed using 654 SNPs weighted by available betas from summary statistics of the genetic variants associated with BMI from a recent GWAS meta-analysis^60^. Two-stage least squares analysis was used to derive one-sample MR estimates for the effect of BMI on 4034 proteins (3622 unique proteins as some proteins were targeted by more than one SOMAmer). MR analyses were conducted in the 2729 participants with genetic data, BMI, and protein data. MR results provide the average difference in protein in rank normalized SD units per 1 normalized SD (~ 4.7 kg/m) higher BMI.

#### Comparison analyses

The effects of caloric restriction and bariatric surgery-induced weight loss were compared to identify consistencies in signal. Results were combined by restricting both results to unique UniProt IDs, then merging DiRECT and By-Band-Sleeve results based on the UniProt ID of the protein^61^. Consistency was determined by effect estimates having the same direction of effect and the corresponding p-values passing pre-specified thresholds in both studies. Opposing effects were defined as estimates displaying opposite directions of effects (for example, the protein levels were raised with one intervention but reduced with the other) and corresponding p-values passing the pre-specified thresholds. Proteins were categorised as only being associated with one intervention where the p-value only passed the pre-specified threshold in one study. Differing effects or null effects in only one study could point towards intervention-specific effects. As proteins were measured using different technologies in each trial, we also explored whether it is appropriate to compare estimates derived using SomaLogic and Olink. For this, we used published correlation information from Haslam et al. to explore how well protein measurements correlate across platforms^32^. Haslam et al. calculated Spearman’s correlation coefficients for every protein that was detected in both platforms, along with the 95% confidence intervals. We integrated these correlation results with our results from each intervention to aid interpretation. For example, the correlation data can help infer whether discordant results are likely due to biological effects specific to the intervention and/or study sample, or whether the differences may be arising due to technological differences across platforms, such as the technologies picking up different isoforms or variants of the same protein^32^. References made herein to ‘correlations across platforms’ refer to these published estimates^32^.

Proteins with consistent or opposite effects across interventions were compared with published MR results in INTERVAL again using the UniProt ID to merge the information. As we used the MR results to look up pre-specified protein results, we deemed MR estimates as putatively causal effects if the 95% confidence intervals did not cross the null (p < 0.05). Proteins which had evidence for a consistent direction of effect across all three study designs were explored as drug targets by searching on DrugBank^33^ and using published drug target data from Finan et al.^34^. We determined if the protein is currently (or has the capability to be) targeted by a drug and extracted what currently approved drugs are used for. This was performed as one way of exploring the possible role of the protein in health and disease.

## Data and code availability

The R code for main analyses presented in this manuscript has been made publicly available on GitHub at https://github.com/lucygoudswaard/Proteome_comparison_paper. The terms of participant consent in DiRECT does not allow making the study data freely available in its raw form. The data used for analysis will be placed on a research data repository (https://researchdata.gla.ac.uk/) with access given to researchers subject to appropriate data sharing agreements. For the By-Band-Sleeve trial, following publication of the main trial results, anonymised individual patient data will be made available upon request to the chief investigator for secondary research, conditional on assurance from the secondary researcher that the proposed use of the data is compliant with the Medical Research Council Policy on Data Sharing regarding scientific quality, ethical requirements, and value for money, and is compliant with the National Institute for Health and Care Research policy on data sharing. A minimum requirement with respect to scientific quality will be a publicly available prespecified protocol describing the purpose, methods, and analysis of the secondary research (e.g., a protocol for a Cochrane systematic review), approved by a UK Research Ethics Committee or other similar, approved ethics review body. Participant identifiers will not be passed on to any third party.

### Supplementary Information

Supplementary Information.

## References

[1] Williams SA (2019). Plasma protein patterns as comprehensive indicators of health. Nat. Med..

[2] Timpson NJ (2011). C-reactive protein levels and body mass index: elucidating direction of causation through reciprocal Mendelian randomization. Int. J. Obes. (Lond.).

[3] Füzéry AK, Levin J, Chan MM, Chan DW (2013). Translation of proteomic biomarkers into FDA approved cancer diagnostics: Issues and challenges. Clin. Proteom..

[4] Malik P (2021). Biomarkers and outcomes of COVID-19 hospitalisations: Systematic review and meta-analysis. BMJ Evid. Based Med..

[5] Khan SS (2018). Association of body mass index with lifetime risk of cardiovascular disease and compression of morbidity. JAMA Cardiol..

[6] Garg SK, Maurer H, Reed K, Selagamsetty R (2014). Diabetes and cancer: Two diseases with obesity as a common risk factor. Diabetes Obes. Metab..

[7] Kortt M, Baldry J (2002). The association between musculoskeletal disorders and obesity. Aust. Health Rev..

[8] Lawlor DA, Tilling K, Davey Smith G (2016). Triangulation in aetiological epidemiology. Int. J. Epidemiol..

[9] Gloy VL (2013). Bariatric surgery versus non-surgical treatment for obesity: a systematic review and meta-analysis of randomised controlled trials. BMJ.

[10] Geyer PE (2016). Proteomics reveals the effects of sustained weight loss on the human plasma proteome. Mol. Syst. Biol..

[11] Figarska SM (2020). Proteomic profiles before and during weight loss: Results from randomized trial of dietary intervention. Sci. Rep..

[12] Oller Moreno S (2018). The differential plasma proteome of obese and overweight individuals undergoing a nutritional weight loss and maintenance intervention. Proteom. Clin. Appl..

[13] Piening BD (2018). Integrative personal omics profiles during periods of weight gain and loss. Cell Syst..

[14] Carayol J (2017). Protein quantitative trait locus study in obesity during weight-loss identifies a leptin regulator. Nat. Commun..

[15] Bruderer R (2019). Analysis of 1508 plasma samples by capillary-flow data-independent acquisition profiles proteomics of weight loss and maintenance. Mol. Cell Proteom..

[16] Wewer Albrechtsen NJ (2018). Plasma proteome profiling reveals dynamics of inflammatory and lipid homeostasis markers after Roux-En-Y gastric bypass surgery. Cell Syst..

[17] Culnan DM, Cooney RN, Stanley B, Lynch CJ (2009). Apolipoprotein A-IV, a putative satiety/antiatherogenic factor, rises after gastric bypass. Obesity (Silver Spring).

[18] Shah RV (2019). Proteins altered by surgical weight loss highlight biomarkers of insulin resistance in the community. Arterioscler. Thromb. Vasc. Biol..

[19] Yousri NA (2022). Proteome-wide associations with short- and long-term weight loss and regain after Roux-en-Y gastric bypass surgery. Obesity (Silver Spring).

[20] Goudswaard LJ (2021). Effects of adiposity on the human plasma proteome: Observational and Mendelian randomisation estimates. Int. J. Obes. (Lond.).

[21] Zaghlool SB (2021). Revealing the role of the human blood plasma proteome in obesity using genetic drivers. Nat. Commun..

[22] Burgess S, Butterworth A, Thompson SG (2013). Mendelian randomization analysis with multiple genetic variants using summarized data. Genet. Epidemiol..

[23] Lawlor DA, Harbord RM, Sterne JA, Timpson N, Davey Smith G (2008). Mendelian randomization: Using genes as instruments for making causal inferences in epidemiology. Stat. Med..

[24] Freeman G, Cowling BJ, Schooling CM (2013). Power and sample size calculations for Mendelian randomization studies using one genetic instrument. Int. J. Epidemiol..

[25] Sun, B. B. *et al.* Genetic regulation of the human plasma proteome in 54,306 UK Biobank participants. *bioRxiv*, 2022.2006.2017.496443 (2022). 10.1101/2022.06.17.496443

[26] Ferkingstad E (2021). Large-scale integration of the plasma proteome with genetics and disease. Nat. Genet..

[27] Kernan WN, Viscoli CM, Makuch RW, Brass LM, Horwitz RI (1999). Stratified randomization for clinical trials. J. Clin. Epidemiol..

[28] McGuire H (2016). Management of type 2 diabetes in adults: summary of updated NICE guidance. BMJ.

[29] Paul S, Thomas G, Majeed A, Khunti K, Klein K (2012). Women develop type 2 diabetes at a higher body mass index than men. Diabetologia.

[30] Douglas IJ, Bhaskaran K, Batterham RL, Smeeth L (2015). Bariatric surgery in the United Kingdom: A cohort study of weight loss and clinical outcomes in routine clinical care. PLoS Med.

[31] B. B. S. C, Group (2023). Roux-en-Y gastric bypass, gastric banding, or sleeve gastrectomy for severe obesity: Baseline data from the By-Band-Sleeve randomized controlled trial. Obesity (Silver Spring).

[32] Haslam, D. E. *et al.* Stability and reproducibility of proteomic profiles in epidemiological studies: comparing the Olink and SOMAscan platforms. *Proteomics*, e2100170 (2022). 10.1002/pmic.202100170 10.1002/pmic.202100170 PMC9923770 35598103

[33] Wishart DS (2006). DrugBank: A comprehensive resource for in silico drug discovery and exploration. Nucl. Acids Res..

[34] Finan C (2017). The druggable genome and support for target identification and validation in drug development. Sci. Transl. Med..

[35] Fachim HA (2021). Relationship between the plasma proteome and changes in inflammatory markers after bariatric surgery. Cells.

[36] Jüllig M (2014). Lower fetuin-A, retinol binding protein 4 and several metabolites after gastric bypass compared to sleeve gastrectomy in patients with type 2 diabetes. PLoS One.

[37] Alfadda AA (2014). A proteomic analysis of excreted and circulating proteins from obese patients following two different weight-loss strategies. Exp. Biol. Med. (Maywood).

[38] Rajpathak SN (2009). The role of insulin-like growth factor-I and its binding proteins in glucose homeostasis and type 2 diabetes. Diabetes Metab. Res. Rev..

[39] Haywood NJ, Slater TA, Matthews CJ, Wheatcroft SB (2019). The insulin like growth factor and binding protein family: Novel therapeutic targets in obesity & diabetes. Mol. Metab..

[40] Faramia J (2021). IGFBP-2 partly mediates the early metabolic improvements caused by bariatric surgery. Cell Rep. Med..

[41] Arend WP, Malyak M, Guthridge CJ, Gabay C (1998). Interleukin-1 receptor antagonist: Role in biology. Annu. Rev. Immunol..

[42] Møller HJ (2012). Soluble CD163. Scand. J. Clin. Lab. Invest..

[43] Lee CH (2019). Serum E-selectin concentration is associated with risk of metabolic syndrome in females. PLoS One.

[44] Ghodsian N (2021). Electronic health record-based genome-wide meta-analysis provides insights on the genetic architecture of non-alcoholic fatty liver disease. Cell Rep Med.

[45] Ritchie, S. C. *et al.* Integrative analysis of the plasma proteome and polygenic risk of cardiometabolic diseases. *bioRxiv*, 2019.2012.2014.876474 (2021). 10.1101/2019.12.14.876474 10.1038/s42255-021-00478-5 PMC8574944 34750571

[46] Fagerberg L (2014). Analysis of the human tissue-specific expression by genome-wide integration of transcriptomics and antibody-based proteomics. Mol. Cell Proteom..

[47] Sethi, A., Raj, A., Wright, K. & Melamud, E. Plasma Proteomic Determinants of Common Causes of Mortality. *PREPRINT (version 1) available at Research Square* (2023). 10.21203/rs.3.rs-2626017/v1

[48] Gudmundsdottir V (2020). Circulating protein signatures and causal candidates for type 2 diabetes. Diabetes.

[49] Issaq HJ, Xiao Z, Veenstra TD (2007). Serum and plasma proteomics. Chem. Rev..

[50] Wik L (2021). Proximity extension assay in combination with next-generation sequencing for high-throughput proteome-wide analysis. Mol. Cell Proteom..

[51] Lean ME (2018). Primary care-led weight management for remission of type 2 diabetes (DiRECT): An open-label, cluster-randomised trial. Lancet.

[52] Lean M (2013). Feasibility and indicative results from a 12-month low-energy liquid diet treatment and maintenance programme for severe obesity. Br. J. Gen. Pract..

[53] Rohloff JC (2014). Nucleic acid ligands with protein-like side chains: Modified aptamers and their use as diagnostic and therapeutic agents. Mol. Ther. Nucl. Acids.

[54] SomaLogic. SomaScan® v4 Data Standardization and File Specification Technical Note [White paper]. (2022). https://somalogic.com/tech-notes/.

[55] Hughes DA (2022). metaboprep: An R package for preanalysis data description and processing. Bioinformatics.

[56] Scott NW, McPherson GC, Ramsay CR, Campbell MK (2002). The method of minimization for allocation to clinical trials. A review. Control Clin. Trials.

[57] Rogers CA (2014). The By-Band study: Gastric bypass or adjustable gastric band surgery to treat morbid obesity: Study protocol for a multi-centre randomised controlled trial with an internal pilot phase. Trials.

[58] Paramasivan S (2017). Enabling recruitment success in bariatric surgical trials: pilot phase of the By-Band-Sleeve study. Int. J. Obes. (Lond.).

[59] Olink Proteomics. PEA – a high-multiplex immunoassay technology with qPCR or NGS readout [White paper]. https://www.olink.com/application/pea-a-high-multiplex-immunoassay-technology-with-qpcr-or-ngs-readout-2/ (2020).

[60] Yengo L (2018). Meta-analysis of genome-wide association studies for height and body mass index in ∼700000 individuals of European ancestry. Hum Mol Genet.

[61] Katz DH (2022). Proteomic profiling platforms head to head: Leveraging genetics and clinical traits to compare aptamer- and antibody-based methods. Sci Adv.

